# Forward programming of hiPSCs towards beta-like cells using Ngn3, Pdx1, and MafA

**DOI:** 10.1038/s41598-024-64346-4

**Published:** 2024-06-13

**Authors:** Abiramy Jeyagaran, Max Urbanczyk, Shannon L. Layland, Frank Weise, Katja Schenke-Layland

**Affiliations:** 1 Institute of Biomedical Engineering, Department for Medical Technologies and Regenerative Medicine, Eberhard Karls University Tübingen, 72076 Tübingen, Germany; 2 Department of Women’s Health, Eberhard Karls University, 72076 Tübingen, Germany; 3 NMI Natural and Medical Sciences Institute at the University Tübingen, 72770 Reutlingen, Germany

**Keywords:** Stem-cell differentiation, Regenerative medicine, Stem-cell biotechnology, Tissue engineering, Biomedical engineering

## Abstract

Transplantation of stem cell-derived β-cells is a promising therapeutic advancement in the treatment of type 1 diabetes mellitus. A current limitation of this approach is the long differentiation timeline that generates a heterogeneous population of pancreatic endocrine cells. To address this limitation, an inducible lentiviral overexpression system of mature β-cell markers was introduced into human induced-pluripotent stem cells (hiPSCs). Following the selection of the successfully transduced hiPSCs, the cells were treated with doxycycline in the pancreatic progenitor induction medium to support their transition toward the pancreatic lineage. Cells cultured with doxycycline presented the markers of interest, NGN3, PDX1, and MAFA, after five days of culture, and glucose-stimulated insulin secretion assays demonstrated that the cells were glucose-responsive in a monolayer culture. When cultured as a spheroid, the markers of interest and insulin secretion in a static glucose-stimulated insulin secretion assay were maintained; however, insulin secretion upon consecutive glucose challenges was limited. Comparison to human fetal and adult donor tissues identified that although the hiPSC-derived spheroids present similar markers to adult insulin-producing cells, they are functionally representative of fetal development. Together, these results suggest that with optimization of the temporal expression of these markers, forward programming of hiPSCs towards insulin-producing cells could be a possible alternative for islet transplantation.

## Introduction

Pancreatic islets of Langerhans are the functional cells of the endocrine pancreas responsible for the maintenance of blood glucose homeostasis^1,2^. In type 1 diabetes, the β-cells, the insulin-producing cells, are lost as a result of an autoimmune attack, leading to the dysregulation of blood glucose levels^3,4^. Continuous monitoring of blood glucose levels and administration of appropriate levels of exogenous insulin is the gold standard treatment for many patients suffering from diabetes; however, there remains the risk of over- and/or under-administering the amount of insulin which calls for more endogenous maintenance of blood glucose such as islet transplantation^5^. There have been numerous advances in islet transplantation approaches in recent years to treat patients with type 1 diabetes including protecting transplanted cells from the host’s immune system and utilizing new sources of β-cells to be transplanted^6^. Of great interest are stem cell-derived β-cells that can act as a continuous source of β-cells for the high demand of endocrine replacement therapy.

Recent advances in studying pancreatic development has improved our understanding of what biochemical and biophysical cues are required to signal stem cells to differentiate towards β-cells^6^. Stage-based differentiation using consecutive incorporation and exclusion of growth factors and / or signalling molecules have been successful in differentiating human embryonic stem cells (hESCs) and human induced pluripotent stem cells (hiPSCs) into glucose-responsive insulin-producing β-cells^7,8^. Through the years, there have been updates to the differentiation protocol where similar culture medium recipes are used with slight changes in timing and concentration of certain growth factors, or cell culture platforms (2-dimensional (2D) vs. 3-dimensional (3D))^9–16^. Along with selection of cells presenting pancreatic progenitor markers at the different stages of differentiation, these protocols have been improved to achieve increased differentiation efficiency of stem cells into β-cells over a month-long period, where they could be further matured in vivo in mice or in vitro through 3D cultures in bioreactors^16,17^. This maturation process and expression of maturation markers are extremely important for the glucose-responsive behaviour of β-cells; however, it is time-consuming and can result in a heterogeneous cell population. Interestingly, overexpression of mature cell markers has also been used to direct human pluripotent stem cells (hPSCs) towards a desired lineage. Through overexpression of cell-specific markers in hPSCs, researchers have essentially reprogrammed or directed the differentiation of multiple cell types including hepatocytes^18^, hematoendothelial cells^19^, neurons^20–22^, skeletal myocytes^22^, oligodendrocytes^22^, megakaryocytes^23^, microglia^24^, and endothelial cells^25,26^. This suggests that similar methods can be used to improve the differentiation efficiency of hiPSCs into β-cells.

Extensive investigation of pancreas development in mice allowed for the identification of different combinations of transcription factor expression required for the specification of various pancreatic cells^27,28^. Interestingly, the adenoviral transfection of three specific transcription factors, NGN3, PDX1, and MAFA were sufficient to reprogram mouse^29^ and rat^30,31^ pancreatic exocrine tissues to insulin-producing β-cells in vivo. Furthermore, this was shown to be sufficient in preventing hyperglycemic episodes in diabetic mice. A similar approach was also successful in reprogramming murine hepatocytes such that they expressed the three markers as well as insulin and were capable of restoring glucose homeostasis in diabetic mice^32–34^. Interestingly, expression of PDX1 and MAFA in murine α-cells was also found to be sufficient to reprogram them into insulin-producing β-cells *in vivo*^35,36^. The expression of these three genes has also been shown to improve differentiation efficiency of mouse ESCs^37^ and hiPSCs^38^ when introduced at the pancreatic progenitor stage of the differentiation protocol in a temporal manner. These studies showed the exogenous expression of NGN3, PDX1, and MAFA were sufficient to reprogram adult pancreatic and hepatic cells into glucose-responsive insulin-producing β-cells. In this study, we investigated whether the expression of NGN3, PDX1, and MAFA in hiPSCs could drive the differentiation of the cells towards the pancreatic lineage within a two-week period. We aimed to determine whether this induced expression of the mature markers can activate and regulate the endogenous expression of the pancreatic and mature β-cell markers. We generated inducible lentiviral constructs for the expression of NGN3, PDX1, and MAFA, to be introduced into hiPSCs and induced the cells in medium supporting pancreatic lineage differentiation with factors known to support β-cell differentiation. Our markers of interest along with two other pancreatic progenitor markers, *NKX6.1* and *MAFB*, were upregulated upon five days of induction. Static glucose-stimulated insulin secretion assays demonstrated that the cells were glucose responsive to levels similar to previously published protocols; however, the upregulation of the gene expression was not directly translated into protein expression upon extended culture periods and endogenous insulin production was not observed. These results demonstrate that the overexpression of the three β-cell markers in hiPSCs over a ten-day culture period is not sufficient for the generation of pancreatic cells. The forward-programming of hiPSCs into mature functional β-cells would be a significant step towards the scaling and commercialization of insulin-secreting cells for transplantation into patients suffering from diabetes. This work serves as a tool to better understand the importance of temporal expression patterns of these three genes and how the inducible lentiviral construct could be optimized to fine-tune their expression to support development towards the pancreatic lineage.

## Methods

### Generation of inducible lentiviral constructs

For the inducible expression of our markers of interest, two lentiviral constructs were generated. The first lentiviral construct, pLenti_EF1a-rtTA_BsdR was generated as follows: The sequence for EF1a-rtTA, flanked with *att*B1 and *att*B2 sites, was synthesized and subsequently shuttled into a pDONR221 backbone through ThermoFisher, yielding an entry vector. Gateway Cloning was then used between this construct and pLenti6.3_V5-DEST_promoterless_mcs_BsdR conferring Blasticidin resistance to generate pLenti_EF1a-rtTA_BsdR.

**Figure 1:**
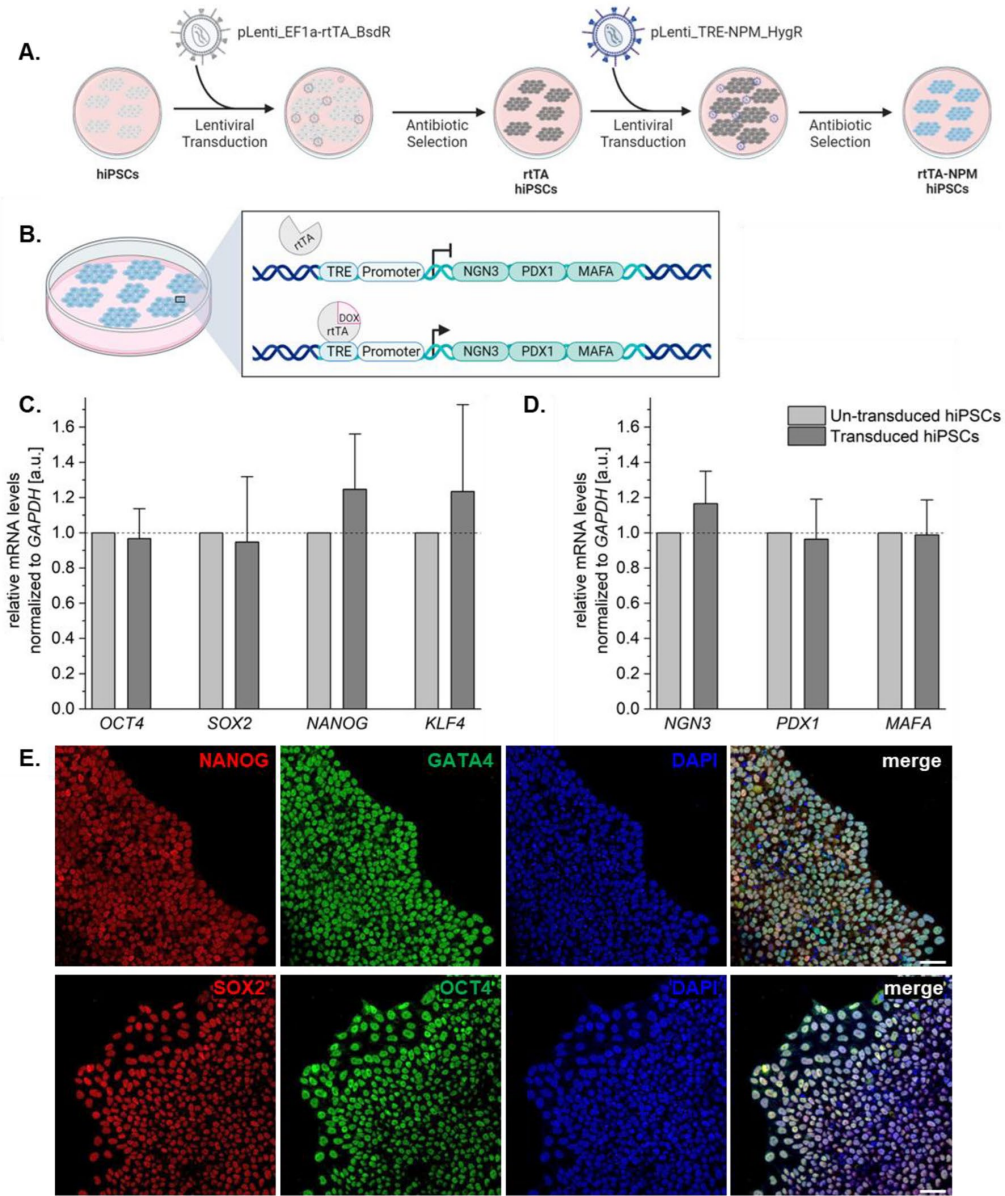
Introduction of inducible lentiviral construct into hiPSCs does not affect pluripotency marker expression. (**A**) Schematic of how the rtTA-NPM hiPSCs were generated and (**B**) how DOX treatment would activate transcription of the markers of interest. Gene expression analysis of transduced hiPSCs for (**C**) stem cell markers and (**D**) markers of interest before DOX treatment relative to un-transduced hiPSCs (dashed line at 1), normalized to GAPDH levels. Unpaired t-test (N = 3). Error bars represent standard deviation. (**E**) IF staining in transduced hiPSCs to confirm stem cell marker expression at the protein level. Scale bar equals 50 µm. Schematics created with BioRender.com.

The second lentiviral construct, pLenti_TRE-NPM_HygR, was generated as follows: The markers of interest, *NGN3* (GenBank ID 50,674), *PDX1* (GenBank ID 3651), and *MAFA* (GenBank ID 389,692) were designed downstream of the TRE3G promoter. Multicistronic expression of the markers of interest was achieved using the self-cleaving 2A-peptides T2A and P2A. This sequence was synthesized through ThermoFisher within a pENTR221 backbone and recombined with pLenti6.3_V5-DEST_promoterless_mcs_HygR conferring HygromycinB resistance to generate pLenti_TRE-NPM_HygR. Final construct maps (Supp.Fig. 1a,c) and detailed cloning information are available upon request.

### Lentivirus production and transduction

The lentiviruses were produced separately using human embryonic kidney 293 T cells. Cells were cultured in DMEM + GlutaMax (Gibco, 31,966–021), 10% FCS (Gibco, 10,270–106), 1% NEAA (Gibco, 11,140–035), 1% L-Glutamine (Gibco, 25,030–024) and 1% Pen/Strep (Gibco, 15,140,122). At 80% confluency, the cells were transfected overnight with the lentiviral constructs along with Lipofectamine 2000 Reagent (Invitrogen, 11,668–500) and Ready-to-use Lentiviral Packaging Plasmid Mix (Cellecta, CPCP2KA) in OptiMEM + GlutaMax (Gibco, 51,985–026), 5% FCS, and 1% Pen/Strep. Medium was changed the next day, and viruses were harvested each day for the next two days and stored at − 80 C. The virus was then centrifuged at 19,600 rpm at 4 °C for 80 min. Supernatant was removed and 100 µL of DPBS + 1% BSA was pipetted on top of the pellet. The tube was then sealed with parafilm and incubated at 4 °C overnight before the pellet was resuspended, and aliquoted. The lentiviral titre was determined using a p24 ELISA.

hiPSCs^39^ were maintained on Matrigel (Corning, 356,231)-coated dishes in mTesR + (StemCell Technologies, 100–1130) medium supplemented with 1% Pen/Strep, and 50.0 µg/mL Normocin (InvivoGen, ant-nr-1). hiPSC were transduced using the EF1a-rtTA_BsdR lentivirus, and successful recombinants were selected for using 1.0 µg/mL of Blasticidin S HCl (Gibco, A1113902) for five days, now referred to as rtTA-hiPSCs. Subsequently, rtTA-hiPSCs were transduced using the TRE-NPM_HygR lentivirus, and successful recombinants were selected for using 25.0 µg/mL of HygromycinB (Gibco, 10,687,010) for six days, now referred to as rtTA-NPM hiPSCs. The rtTA-NPM hiPSCs were maintained on Matrigel-coated dishes in mTesR + medium supplemented with 1.0 µg/mL Blasticidin, 25.0 µg/mL HygromycinB, 1% Pen/Strep, and 50.0 µg/mL Normocin.

### Doxycycline induction towards the pancreatic lineage

To begin the induction towards the pancreatic lineage, rtTA-NPM hiPSCs were cultured in medium supporting β-cell differentiation^14^ with and without 1.0 µg/mL doxycycline hyclate (Sigma Aldrich, D9891-1G). The medium recipe is as follows: MCDB131 (Cellgro, 15–100-CV), 3.6 mg/mL D-( +)-Glucose (Sigma, G7528), 1.75 mg/mL NaHCO_3_ (Sigma, S5761), 20.0 mg/mL FAF-BSA (Sigma, A9576), 1% Glutamax (LifeTech, 35,050–061), 0.5% ITS-X (Invitrogen, 51,500,056), 0.25 mM Vitamin C (Sigma, A4544), 100 nM Retinoic Acid (Sigma, R2625), 1.0 µM T3 (EMD Millipore, 64,245), 20.0 ng/mL Betacellulin (Peprotech, 100–50), 10.0 µM Y27632 (Abcam, ab120129), 0.25 µM Sant1 (Sigma S4572), 10.0 µg/mL Heparin (Sigma H3149), 10.0 µM Alk5 II (Axxora ALX-270–445), 1.0 µM XXI (EMD Millipore 565,790), 1% Pen/Strep, and 50.0 µg/mL Normocin.

During the induction, cells were cultured for five days as a monolayer with daily medium changes. At the end of the five days, 3D spheroids were generated by creating a single cell suspension of the cultures and seeding 2000 cells / well in a 96-well u-bottom plate (Greiner Bio-One, 650,101). The spheroids were cultured for five more days without further medium changes for a total culture period of ten days. All results presented are from at least three separate inductions.

### RNA extraction and RT-qPCR

The Qiagen RNeasy Mini Kit (Qiagen, 74,101) was used for RNA extraction of samples and DNase (Promega, M6101) treatment was performed according to manufacturer’s protocol. cDNA synthesis was performed using M-MuLV Reverse Transcriptase (New England Biolabs, M0253S), and qPCR was run using the Universal PCR Master Mix (Applied Biosystems, 4,304,449). The list of primers used can be found in Supplementary Table 1. Ready-to-use mixtures of primers and probes (Gene Expression Assays, Thermo Fisher, 4,331,182) were used unless otherwise specified. Gene expression was normalized to the housekeeper gene *GAPDH.* Relative mRNA levels (2^ΔΔCt) of the transduced rtTA-NPM hiPSCs were determined in relation to the un-transduced hiPSCs which was represented with a value of 1. Fold change of mRNA levels (ΔΔCt) of the cells cultured with and without DOX were determined in relation to that of the rtTA-NPM hiPSCs at the start with a timepoint 0 (TP0).

### Ethical statement for the study of human tissue

Studies with human fetal tissue and human adult donor islets were conducted with approval from the Ethics Committee at the Medical Faculty of the Eberhard Karls University Tübingen and at the University Hospital in Tübingen in accordance with the ICH-GCP guidelines (IRB #406/2011BO1 and #290/2016BO1). Human islets for research were provided by the Alberta Diabetes Institute IsletCore at the University of Alberta in Edmonton (www.bcell.org/adi-isletcore). Islet isolation was approved by the Human Research Ethics Board at the University of Alberta (Pro00013094). All donors' families gave informed consent for the use of pancreatic tissue in research.

### Human donor islet culture and static GSIS

Healthy human donor islets were purchased from the Alberta Diabetes Institute IsletCore (Alberta, Canada). Individual donor islets were handpicked into wells of 96well U-bottom non-adherent well plates (Greiner Bio-One, 650,101) and cultured for three days to recover from shipping conditions in CMRL1066 without Glutamine (Gibco, 21,530–027), 0.5% FAF-BSA (Sigma Aldrich, A9576), 1 g/L glucose (Gibco, A24940-01), 4 mM Glutamax (Gibco, 35,050–061), 1% Pen/Strep (Gibco, 15,070–063), and 100 µg/mL Normocin (InvivoGen, ant-nr-1).

For the static GSIS assays, donor islets were incubated for one hour in β-Krebs with 2 mM glucose. Donor islets were then incubated in β-Krebs buffer with either 2 mM glucose or 20 mM glucose for another hour, following which the supernatant was collected and stored at -20 °C. The donor islets were lysed with acid ethanol overnight at 4 °C, collected, and stored at -20 °C for insulin content analysis. The levels of insulin secreted by the donor islets and the insulin content within the donor islets were analyzed using the human insulin ELISA kit (Mercodia, 10–1132-01). The GSIS index was calculated by dividing each samples’ insulin secretion during 20 mM glucose by 2 mM glucose.

### Immunofluorescence (IF) staining and quantification

Following the five-day monolayer culture, cells were prepared for IF staining of the markers of interest. Cells were washed twice with PBS, fixed in 4% PFA for 20 min at room temperature (RT), permeabilized with 1% Triton-X for 30 min at RT, blocked with Goat Block for 30 min at RT, and incubated with the primary antibody overnight at 4 °C (see Supplementary Table 2 for list of antibodies used). Cells were washed and first incubated with the secondary antibody for 30 min at RT in the dark, and then with 2 µg/ml of 4′,6-Diamidin-2-phenylindole (DAPI) solution for 10 min at RT in the dark. Samples were mounted with Prolong Gold Antifade Mountant (Thermo Fisher Scientific, P36930) and imaged using the laser scanning microscope 780 (Carl Zeiss GmbH, Jena, Germany).

The 10-day spheroids, adult donor islets (from the McDonald Laboratory, Alberta, Canada), and 10-week-old fetal tissues (from the University of Tübingen, Tübingen, Germany) were prepared for IF staining, as previously described^40^. Briefly, samples were washed with PBS, fixed in 4% PFA, and embedded in paraffin with a Shandon Citadel 1000 (Thermo Fisher Scientific, Waltham, MA, USA). Samples were sectioned into 3 µm sections (Microtome RM2145, Leica, Nussloch, Germany), and deparaffinized with xylene, graded ethanol (100−50%), and VE-water. Antigen retrieval was performed using both Tris–EDTA (pH 9.0) and Citrate (pH 6.0) buffers. Primary antibody incubation was performed overnight at 4 °C, followed by secondary antibody incubation for 30 min at RT in the dark, with either DAPI solution for 10 min or DRAQ5 (ThermoFisher, 62,251) for 30 min at RT in the dark for nuclear staining. Samples were treated with the Vector® TrueVIEW® Autofluorescence Quenching Kit (Vector Labs, SP-8400) for 1 min at RT before mounting with Prolong Gold Antifade Mountant. Images were obtained using the laser scanning microscope 780. Positive nuclear staining of the markers of interest was manually counted and normalized per DAPI or DRAQ5 nuclear count. Gray value intensity (GVI) was determined per channel for each image, subtracted by the value for the negative control, and then normalized per DAPI or DRAQ5 nuclear count.

### Glucose-stimulated insulin secretion (GSIS) assays

GSIS assays were performed in β-Krebs buffer (recipe can be found in Supplementary Table 3) supplemented with glucose. Cells were washed with PBS and synchronized in β-Krebs buffer with 2 mM glucose (low glucose condition) for one hour in the incubator. Following the synchronization step, cells were either incubated in β-Krebs buffer with either 2 mM glucose or 20 mM glucose (high glucose condition) for one hour in the incubator for the static GSIS assays. For dynamic GSIS assays, the spheroids were incubated in β-Krebs buffer with either 2 mM glucose for an hour followed by buffer with 20 mM glucose for one hour, and then again in buffer with 2 mM glucose for an hour in the incubator. Supernatant was then collected at the end of each incubation and stored at -20 °C overnight before performing either an insulin ELISA (Mercodia, 10–1132-01) or C-peptide ELISA (Mercodia, 10–1141-01). Cells were then trypsinized and counted for normalization of insulin secretion per cell count or per spheroid size.

### Statistics

Data presented is collected from at least three separate induction experiments where the data is presented as mean ± standard deviation (s.d.). Outliers were identified with Grubb's test (*p* ≤ 0.05). Unpaired t-tests were used to analyze statistical differences between groups.

## Results

### hiPSCs maintain their stem cell characteristics following transduction of inducible lentiviral constructs

**Figure 2:**
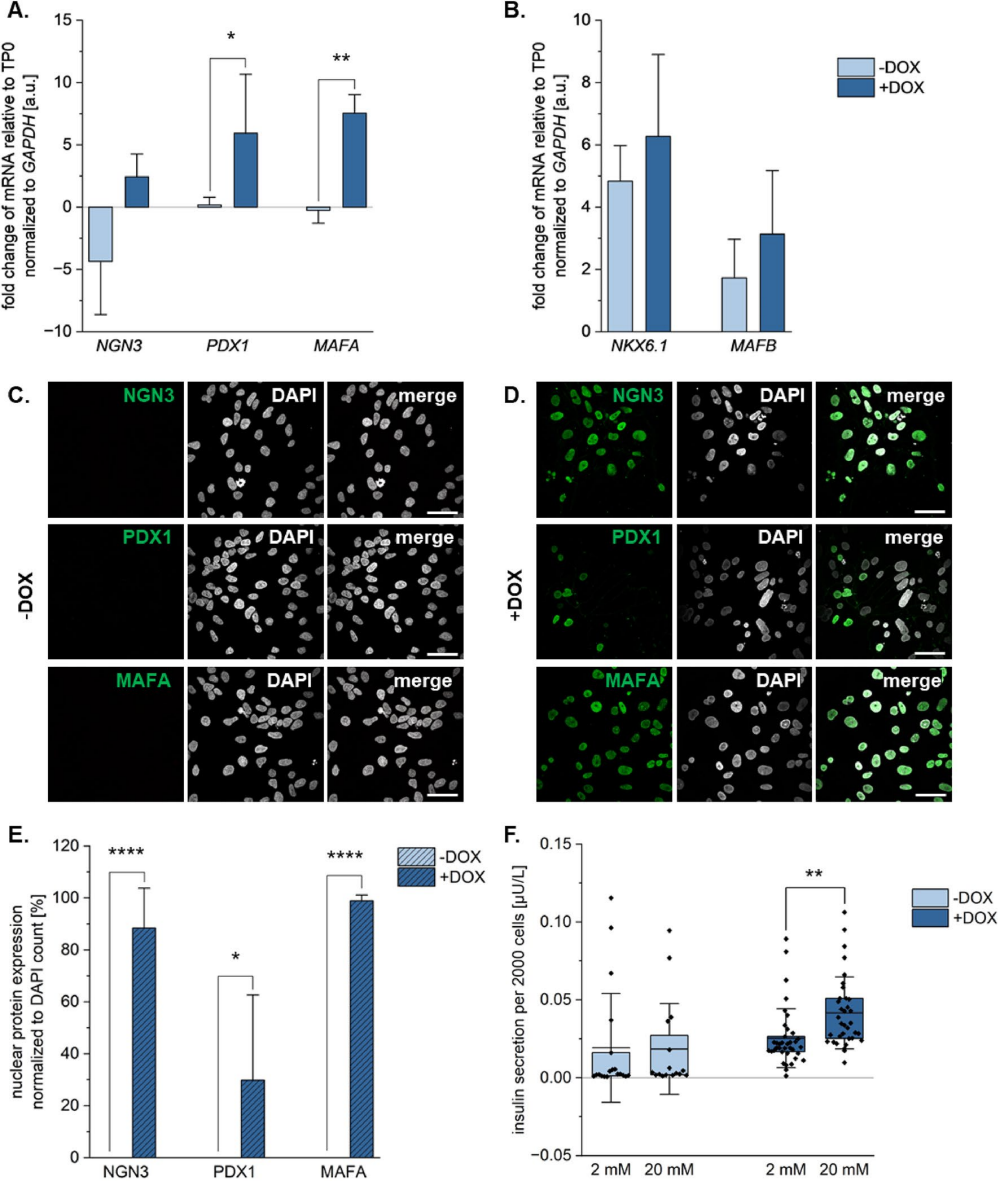
Markers of interest are upregulated at the gene and protein level upon five days of DOX treatment as a monolayer. Gene expression analysis of (**A**) markers of interest and (**B**) pancreatic progenitor markers Nkx6.1 and MafB after 5 days of culture with and without DOX. All values normalized to GAPDH and relative to timepoint 0 (TP0) at the stem cell stage. IF staining for markers of interest in cells cultured for five days (**C**) without and (**D**) with DOX. (**E**) Quantification of the % nuclear protein expression of the markers of interest, normalized to DAPI counts. Scale bar equals 50 µm. (**F**) Amount of insulin secreted upon stimulation with 2 mM and 20 mM glucose, normalized to cell count, during the static GSIS. Unpaired t-test (N = 3, n ≥ 9), * p ≤ 0.05, ** p ≤ 0.01, **** p ≤ 0.0001. Error bars represent standard deviation.

Following generation of the two lentiviral constructs and transduction of hiPSCs (Fig. 1a,b), successful recombinants were selected for through antibiotic treatments of Blasticidin and Hygromycin B using pre-determined concentrations from the antibiotic kill curves on these cells (Supp.Fig. 1b,d,e). The transduced hiPSCs were then assessed to ensure their naïveness through RT-qPCR of *OCT4*, *SOX2*, *NANOG*, and *KLF4*. All genes were normalized to *GAPDH* and assessed relative to the expression levels of the un-transduced hiPSCs. The rtTA-NPM hiPSCs had similar expression levels of the naïve stem cell markers to the un-transduced hiPSCs (Fig. 1c; unpaired t-test, n = 3, *OCT4*: *p* = 0.7514; *SOX2*: *p* = 0.8178; *NANOG*: *p* = 0.2464; *KLF4*: *p* = 04,572), which was also observed through IF staining for these markers in the rtTA-NPM hiPSCs (Fig. 1e). We also wanted to ensure that the markers of interest, *NGN3*, *PDX1*, and *MAFA* were not prematurely expressed in the absence of doxycycline (DOX). RT-qPCR analysis of these genes showed that there were no significant differences in expression levels between the un-transduced and transduced hiPSCs, suggesting there is no premature expression of the markers of interest at the hiPSC stage (Fig. 1d; unpaired t-test, n = 3, *NGN3*: *p* = 0.1945; *PDX1*: *p* = 0.7954; *MAFA*: *p* = 0.9229). Further, the *rtTA* gene, whose protein product is needed for the inducible expression of the gene expression system was significantly upregulated in our rtTA-NPM hiPSCs compared to the un-transduced hiPSCs (Supp.Fig. 2a) as expected.

### Markers of interest are upregulated at the gene and protein levels upon five days of DOX-treatment

Upon culture in medium supporting pancreatic lineage specification for five days, cells cultured with DOX express greater levels of the markers of interest compared to those cultured without DOX relative to timepoint zero (TP0) when cells were still in their stem cell state (Fig. 2a), where *PDX1* and *MAFA* gene expression levels were significantly increased in cells cultured with DOX than those cultured without DOX (unpaired t-test, n = 3, *PDX1*: *p* = 0.042; *MAFA*: *p* = 0.002). Interestingly, two other pancreatic lineage markers *NKX6.1* and *MAFB* were also upregulated upon five days of culture in the medium with and without DOX compared to day zero (Fig. 2b). The markers of interest were also present at the protein level in the nucleus of significantly more cells in the DOX-treated cultures than in the cultures without DOX (Fig. 2c–e). NGN3 was observed in 88.4% (± 7.4%) of the cells cultured with DOX compared to 0.0% (± 0.0%) of the cells cultured without DOX (unpaired t-test, n ≥ 9, *p* = 3.352*10^−14^). PDX1 was observed in 29.8% (± 15.9%) of the cells compared to 0.0% (± 0.0%) of the cells cultured without DOX (unpaired t-test, n ≥ 9, *p* = 0.013). MAFA was observed in 98.9% (± 1.1%) of the cells cultured with DOX compared to 0.0% (± 0.0%) of the cells cultured without DOX (unpaired t-test, n ≥ 9, *p* = 1.380*10^−33^). Interestingly, markers NGN3 and PDX1 were also observed at the cell membranes and so GVI analysis was also performed to account for the expression of the proteins outside of the nucleus. Similar trends were observed when looking at the GVI of the marker expression where the cultures without DOX had expression patterns similar to the negative control and the DOX-treated cultures had significantly greater GVI expression for all markers (Supp.Fig. 2b). To determine whether the cells show functional responses to glucose, static glucose-stimulated insulin secretion (GSIS) assays were performed. While the cultures without DOX did not show a functional response to glucose stimulation (2 mM: 0.019 µU/L ± 0.033 µU/L (2 mM) vs. 0.018 µU/L ± 0.028 µU/L (20 mM), unpaired t-test, n ≥ 16, *p* = 0.9479), the five-day cultures with DOX did secrete significantly more insulin at the 20 mM glucose condition than at the 2 mM glucose (Fig. 2f; 0.025 µU/L ± 0.017 µU/L (2 mM) vs. 0.042 µU/L ± 0.023 µU/L (20 mM), unpaired t-test, n ≥ 16, *p* = 0.0014).

### Extended culture as a 3D spheroid maintains NGN3 and MAFA expression, and improves glucose-responsive insulin secretory behaviour

**Figure 3:**
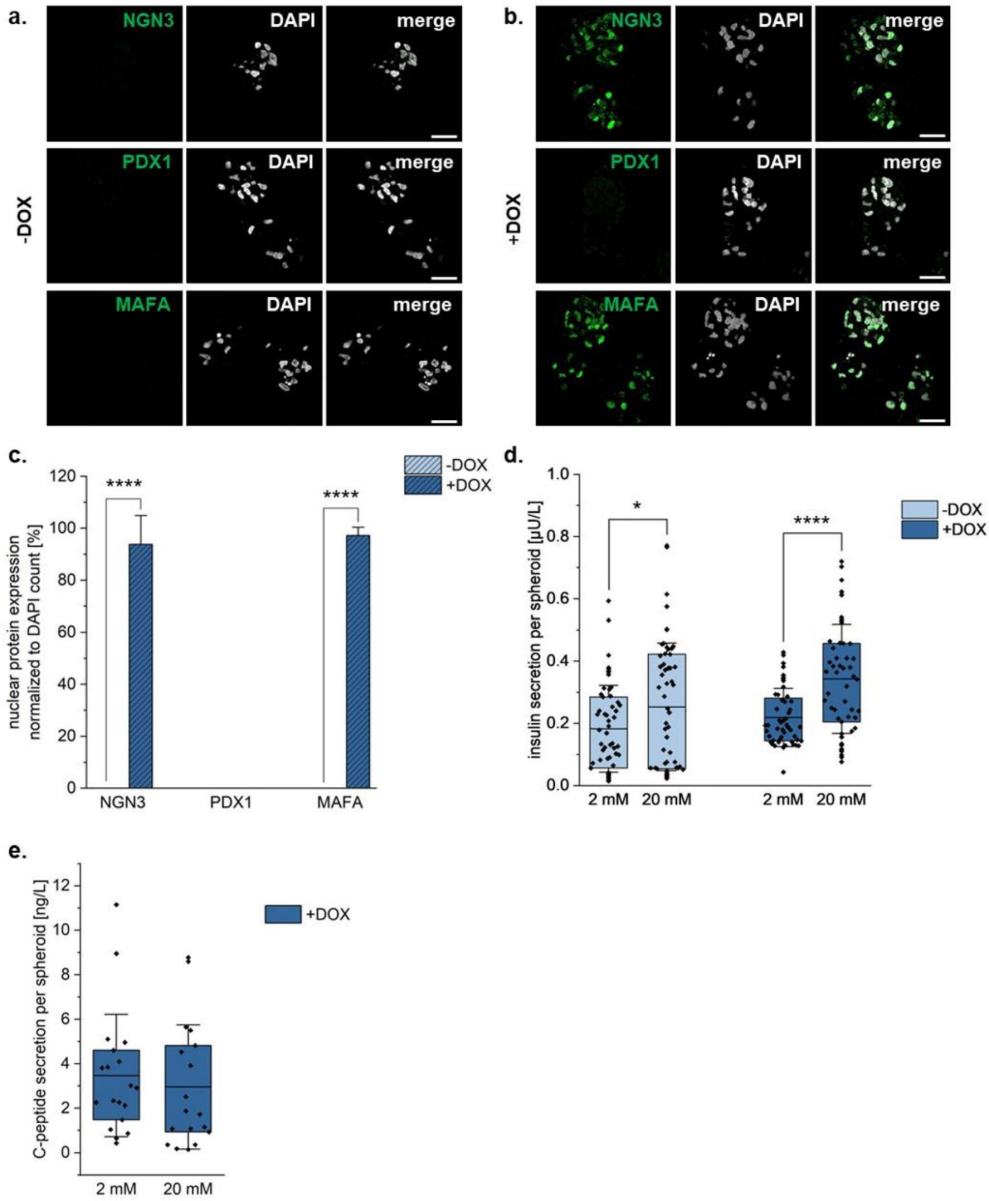
NGN3 and MAFA expression maintained upon ten days of culture as a 3D spheroid. IF staining for markers of interest in cells cultured for ten days with the last five days as a 3D spheroid (**A**) without and (**B**) with DOX. (**C**) Quantification of the % nuclear protein expression of the markers of interest, normalized to DAPI counts. Scale bar equals 25 µm. (**D**) Amount of insulin secreted by cells cultured with DOX upon stimulation with 2 mM and 20 mM glucose, normalized to cell count, during the static GSIS. (**E**) Amount of C-peptide secreted by cells cultured with DOX upon stimulation with 2 mM and 20 mM glucose, normalized to cell count. Unpaired t-test (N = 3, n ≥ 9), * *p* ≤ 0.05, **** *p* ≤ 0.0001. Error bars represent standard deviation.

As human islets of Langerhans are found in clusters, and cell–cell contact has been shown to improve insulin secretion of β-cells ^1^, at day five of culture, the cells were aggregated into spheroids of 2000 cells each and cultured for another five days. At the end of the ten-day culture, NGN3 and MAFA remained to be expressed significantly greater in the cells of spheroids cultured with DOX versus without DOX (Fig. 3a–c: unpaired t-test, n ≥ 9, NGN3: 0.0% ± 0.0%, s.d. (-DOX) vs. 93.8% ± 5.3% (+ DOX), *p* = 3.976*10^−17^; MAFA: 0.0% ± 0.0% (-DOX) vs. 97.2% ± 1.5% (+ DOX), *p* = 1.815*10^−32^). Unexpectedly, in the DOX-treated cultures, nuclear expression of PDX1 was lost at the end of the ten-day culture period with 0.0% (± 0.0%) of cells both with and without DOX (Fig. 3a–c), though gene expression was observed after ten days of culture (Supp.Fig. 3a). To determine whether this could be attributed to differential localization, expression of the proteins through GVI analysis of the IF staining was performed. Again, similar trends were observed where there was a significant increase in NGN3 and MAFA expression in the DOX-treated versus without DOX cultures; however, no significant differences were observed in expression of PDX1 (Supp.Fig. 3b). Static GSIS assays demonstrated that spheroids cultured either with or without DOX secreted significantly more insulin to the increased glucose treatment (Fig. 3d: unpaired t-test, n ≥ 18, -DOX: 0.18 µU/L ± 0.07 µU/L (2 mM) vs. 0.25 µU/L ± 0.10 µU/L (20 mM), *p* = 0.0342; + DOX: 0.22 µU/L ± 0.05 µU/L (2 mM) vs. 0.34 µU/L ± 0.09 µU/L (20 mM), p = 1.776*10^–5^). Unfortunately, the DOX-treated cultures did not show functional responses to multiple glucose stimulations in a dynamic GSIS (Supp.Fig. 3b). C-peptide secretion analysis of the DOX-treated spheroids showed no significant differences in C-peptide secretion between the low and high glucose conditions (Fig. 3e, unpaired t-test, n ≥ 18, + DOX: 3.47 ng/L ± 2.68 ng/L (2 mM) vs. 2.96 ng/L ± 2.71 ng/L (20 mM), *p* = 0.5759). This suggested that the majority of the insulins being secreted at both the five-day monolayer culture and at the ten-day spheroid timepoint is likely uptake of insulin from the medium rather than insulin that the cells produce.

**Figure 4:**
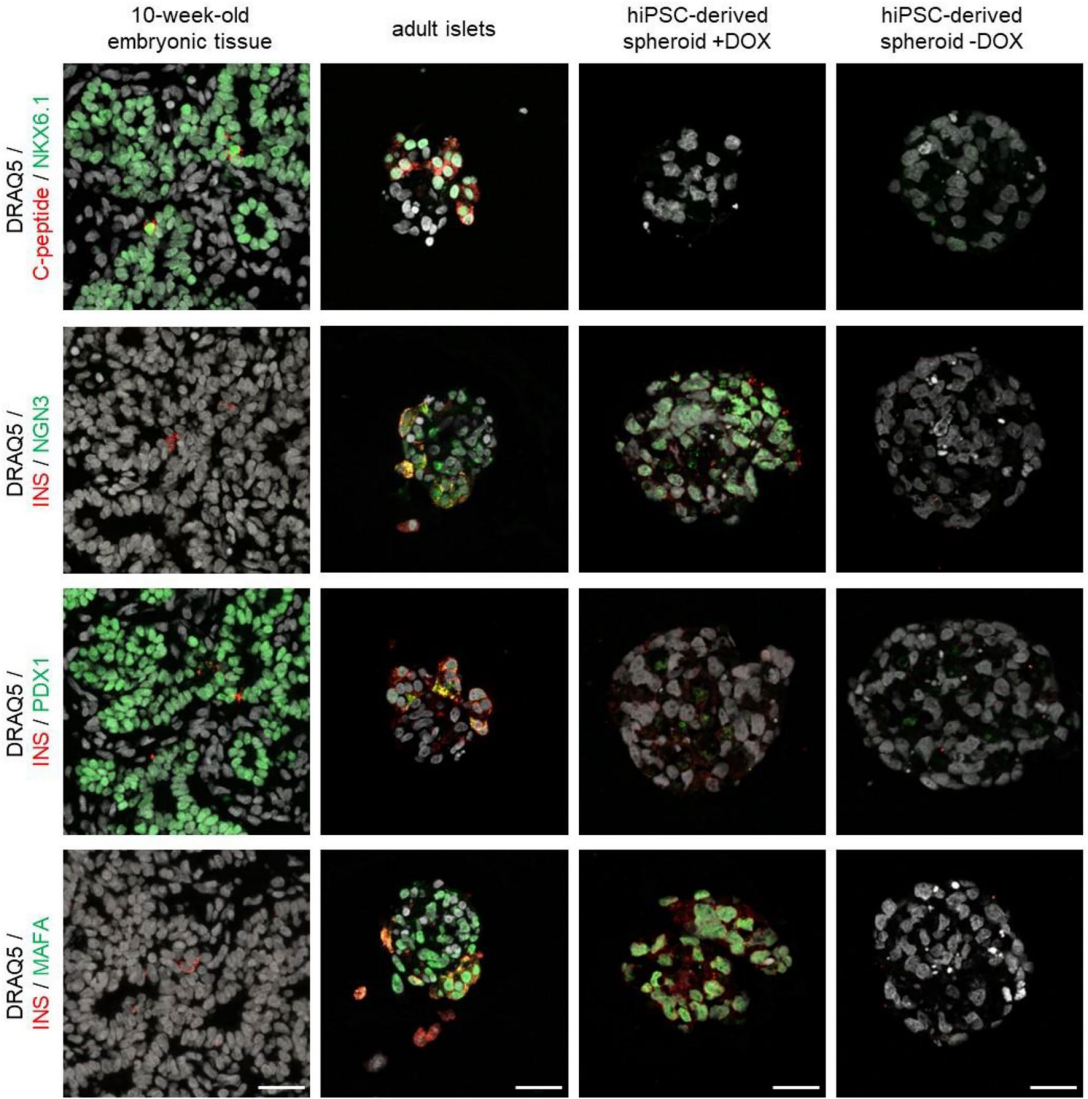
hiPSC-derived spheroids cultured with DOX present similar markers to adult donor islets but are functionally similar to embryonic tissue. 10-week-old embryo tissues, adult donor islets, and the hiPSC-derived spheroids cultured with and without DOX were assessed for protein expression of the three markers of interest (NGN3, PDX1, MAFA), NKX6.1, and the functionality markers C-peptide and insulin (INS). Scale bar equals 25 µm.

To determine how these cells compare to human fetal tissues and human adult islets, IF staining for the markers of interest was performed (Fig. 4). The pancreatic progenitor marker NKX6.1 was observed in the nucleus of the pancreatic cells in the fetal tissues with a limited number of cells showing insulin production through the C-peptide staining. Within the adult donor islets of Langerhans, NKX6.1 was observed in the nucleus of cells that also showed cytoplasmic C-peptide expression. The hiPSC-derived spheroids, both with and without DOX, did not show any presence of NKX6.1 or C-peptide. In 10-week-old fetal tissue, PDX1 was expressed in the nucleus of cells of the pancreas with a limited number of cells also showing cytoplasmic insulin staining. In adult donor islets, PDX1 can be observed to be both nuclear and cytoplasmic with some co-localization with insulin. In our DOX-treated hiPSC-derived spheroids, PDX1 was found to have puncta-like expression within the cytoplasm whereas this is absent in the spheroids without DOX. NGN3 and MAFA were absent in the fetal pancreatic tissues and the hiPSC-derived spheroids without DOX. In adult donor islets, NGN3 was present both in the nucleus and cytoplasm of some cells where it colocalized with the insulin expression. In the hiPSC-derived spheroids with DOX, NGN3 was present in the nucleus of most cells while absent in spheroids cultured without DOX. MAFA was found in the nucleus of cells of the adult donor islets and all the cells of the hiPSC-derived spheroids with DOX, while it was absent in the hiPSC-derived spheroids without DOX. The donor islets assessed were from healthy donors; however, it must be noted that they did not show a functional GSIS response in our culture conditions (Supp.Fig. 4). This could be attributed to donor variability and/or the heterogeneous composition of the donor islets and needs to be considered when assessing the expression patterns of the β-cell markers.

## Discussion

Stem cell-derived β-cells possess great potential to improve efficacy of islet transplantations by overcoming issues regarding immune rejection and availability^6^. Increasing understanding of pancreatic development and the required markers in model organisms and, recently, in human embryos has progressed the field of stem cell-derived β-cells greatly. The developmental process was recapitulated in vitro using hPSCs and chemical signaling through incorporation of various growth factors, supplements, and / or inhibitors in the cell culture medium. Regular changes to the media recipe allowed for the differentiation of the cells towards the pancreatic lineage and eventually to β-cells^6^; however, this process can require up to two months to generate a heterogenous population of pancreatic cells^16^. Past studies have shown that transcription factor overexpression in stem / progenitor cells can result in the differentiation into the desired cell type in a time-effective manner. To address the extended duration and low numbers of differentiated cells from classical differentiation protocols, we transduced hiPSCs with lentiviral constructs that allow for the inducible overexpression of β-cell markers. In this study, we generated hiPSCs with lentiviral constructs for the inducible overexpression of β-cell markers NGN3, PDX1, and MAFA to potentially reduce the required differentiation time and increase the number of mature β-cells produced. Interestingly, the cells cultured with DOX expressed the markers of interest and showed glucose-responsive insulin secretion after five days. When the cells were cultured for a further five days as a 3D spheroid, the expression of NGN3 and MAFA were maintained; however, PDX1 expression was lost. Further assessment of insulin production through C-peptide analysis showed that the cells were not producing insulin themselves, and were rather secreting insulin taken up from the medium. When comparing the hiPSC-derived DOX-treated spheroids to human 10-week-old fetal and adult pancreatic tissues, we found that these cells are still quite immature and require more cues and culture time to differentiate towards the pancreatic lineage. Together, these results demonstrated that the simultaneous overexpression of NGN3, PDX1, and MAFA is not sufficient for the forward programming of hiPSCs towards the pancreatic lineage within a ten-day period.

It has been well observed that the source of the cells used for generation of the hiPSCs, in terms of organ origin and donor’s genetic background, can contribute to the differentiation potential of hiPSCs. During the reprogramming process of somatic cells to hiPSCs, the epigenetic landscape is not necessarily completely remodelled^41–44^. which then influences the lineage towards which the hiPSCs would more readily differentiate. Comparison of gene expression profiles of original somatic cells, the corresponding hiPSC line, and embryonic stem cells showed that genes expressed in the somatic cells were repressed in the corresponding hiPSC line though were found at much lower levels in the ESCs. This suggested that the hiPSCs retained the hypomethylation of the somatic genes, maintaining some of the initial transcriptional landscape^43–45^. The retention of epigenetic landscapes/memory of hiPSCs of the somatic cells they were derived from also explains the discrepancy between hESCs and hiPSCs^41,42^ and/or alteration of metabolic pathways during the reprogramming of somatic cells into hiPSCs^46,47^. Interestingly, it has also been observed that donor-specific differences to have a greater effect on differentiation potential over the tissue of origin^48^ and passage number^49^. Interestingly, a recent study overexpressing the same three transcription factors in adult gut stem cells had promising results following just three weeks of culture^50^. Using similar expression constructs to overexpress NGN3 for the first two days followed by continuous expression of PDX1 and MAFA, Huang et al.^50^ were able to successfully differentiate adult gut stem cells into glucose-responsive insulin-producing cells similar to β-cells and were able to restore glucose homeostasis in diabetic mice for at least 100 days^50,51^. Fontcuberta-PiSunyer, et al.^52^ directly reprogrammed human fibroblasts into glucose-responsive β-cells using a similar ten-day protocol with adenoviral constructs for the exogenous expression of NGN3, PDX1, and MAFA, followed by expression of PAX4 and NKX2.2 in the first week of culture. Both these studies used similar lentiviral constructs and culture conditions as in our study, where they performed monolayer cultures in the first week and then spheroid formation. A key difference from these two protocols compared to ours is the timing of expressing the markers of interest. Rather than expression of all three markers continuously for the entire culture period, Huang et al.^50^ only induced expression of NGN3 for the first two days and then expressed PDX1 and MAFA, while Fontcuberta-PiSunyer et al.^52^ introduced adenoviral constructs for NGN3, PDX1, and MAFA together and separate constructs for PAX4 and NKX2.2 were introduced later in the culture. This suggests that the continuous expression of these markers in our hiPSCs could have resulted in overload of cellular machinery for the continuous protein production. After five days of culture with DOX, NGN3 and MAFA were observed throughout the culture period while PDX1 was lost which could explain the lack of insulin production and glucose-responsive functionality. The loss of PDX1 expression could be a possible mechanism of compensation which could also be attributed to the gene’s position within the lentiviral construct and the vector copy number.

The lentiviral construct design and integration could also have posed an effect on the expression levels of our markers of interest. Following titration of the lentivirus using a p24 ELISA, the virus was transduced into the cells and positively selected for through antibiotic treatment; however, the number of lentiviral constructs introduced into each cell was not controlled for. The positive selection through the antibiotic treatment ensures that there is at least one copy of the integrated into its genome but not that it is equal in each cell^53^. There have been many developments in the field of lentiviral gene therapy especially considering the design of the lentiviral construct including creating only one inducible lentiviral construct with all the necessary components^54,55^, and / or directing the integration of the construct(s) into specific loci such that only one copy is integrated per cell^22^. Adapting the lentiviral design to only have one copy per cell could overcome the issue of PDX1 being lost at the protein level upon longer culture. This could possibly avoid protein compensation^56^ where the cell maintains homeostasis after protein translation and targets proteins to degradation rather than controlling the transcription. Through downregulation of PDX1, the cell prevents differentiation towards the pancreatic lineage; however, as NGN3 and MAFA are maintained, this raises the question whether the position of PDX1 in the lentiviral construct, in the middle of the three markers of interest, would be an issue. The T2A and P2A self-cleaving peptides are widely used for multi-cistronic expression constructs^57–59^. It has been shown that in tri-cistronic constructs similar to ours, there was greatest expression of the first gene, followed by the third gene, and weakest expression of the second gene^60^. This is in line with our results on day five where NGN3 and MAFA, our first and third genes in the construct were present in around 88% and 98% of the cells, respectively, while PDX1, the second gene in the construct, was only present in around 30% of the cells. Introducing separate lentiviral constructs for each gene could be a possible solution; however, the risk of disrupting the genome with three lentiviral constructs could pose a problem in genetic integrity. The constructs for the inducible overexpression of each gene would have to be carefully designed and introduced to the cells to ensure successful integration and reprogramming without negatively impacting the genome.

Recent success of studies^50,52^ using similar lentiviral constructs and culture conditions to reprogram adult (stem) cells into glucose-responsive insulin-producing β-cells provide hope that a few adaptations to our protocol can also achieve such results. Future studies looking to direct the differentiation of hiPSCs towards β-cells should use separate lentiviral constructs with different induction factors for each marker of interest to allow for the most efficient expression of the markers of interest and control over their temporal expression patterns. Multiple hiPSC donors and tissue origins should also be studied to determine whether one tissue or donor is more readily differentiated towards the pancreatic lineage. When considering the use of hiPSC lines, many studies look at somatic cells that are most efficiently reprogrammed; however, it has also been shown that hiPSCs from somatic cells that were more resistant to being reprogrammed may be more effectively differentiated due to their epigenetic memory^61^. Further investigation into extending the culture conditions with the temporal expression of these three genes in hiPSCs has potential in dramatically improving the availability of hiPSC-derived β-cells for therapeutic purposes.

### Supplementary Information

Supplementary Information.
