# Supplementary material for: Forward programming of hiPSCs towards beta-like cells using Ngn3, Pdx1, and MafA
Source: Sci Rep. 2024 Jun 13;14:13608. doi: 10.1038/s41598-024-64346-4 (PMC11176171; doi:10.1038/s41598-024-64346-4)
Supplement: Supplementary file 1 — Supplementary Information. [file 41598_2024_64346_MOESM1_ESM.pptx]

## Slide 1
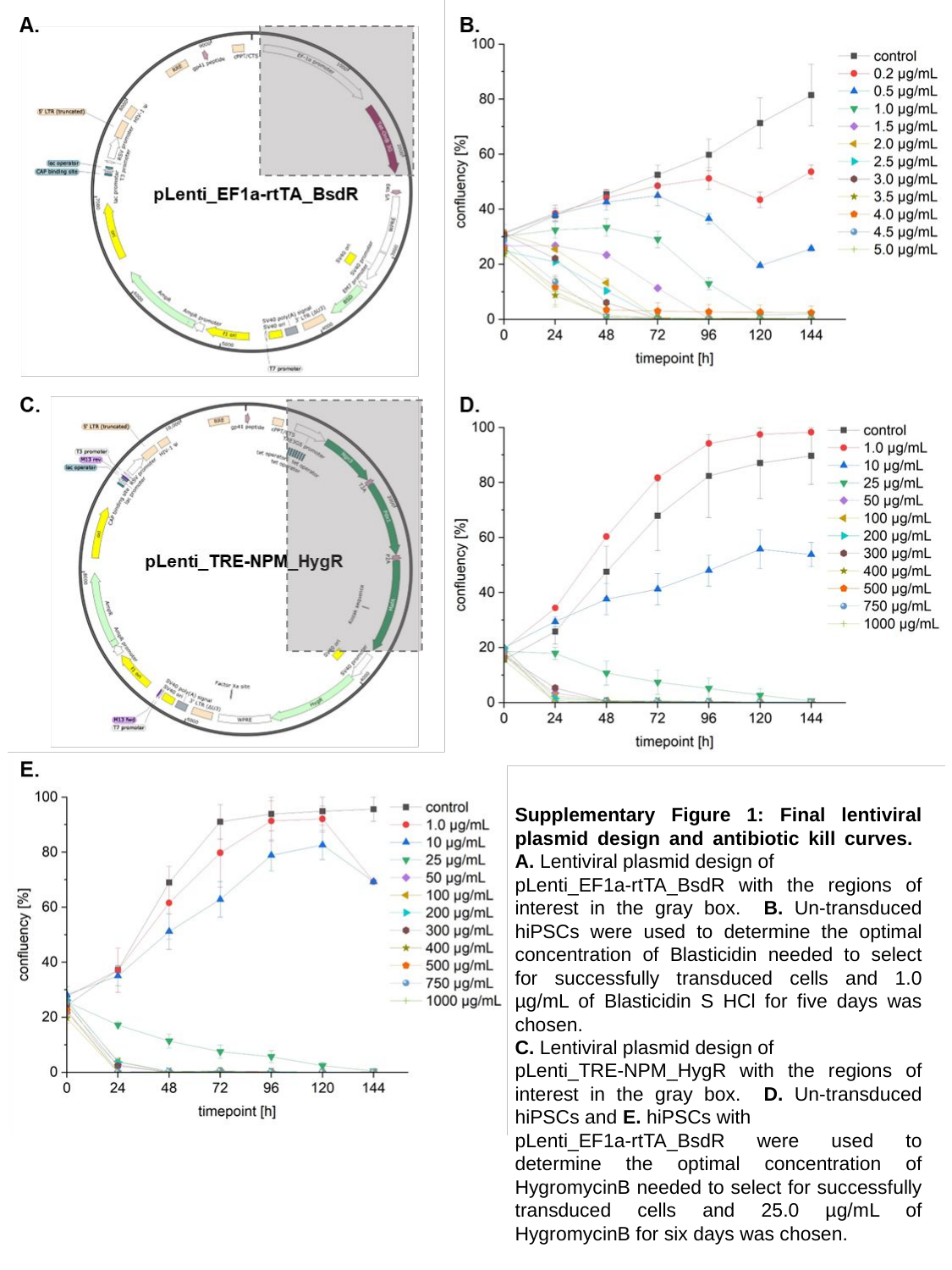

Supplementary Figure 1: Final lentiviral plasmid design and antibiotic kill curves. A. Lentiviral plasmid design of
pLenti_EF1a-rtTA_BsdR with the regions of interest in the gray box. B. Un-transduced hiPSCs were used to determine the optimal concentration of Blasticidin needed to select for successfully transduced cells and 1.0 µg/mL of Blasticidin S HCl for five days was chosen.
C. Lentiviral plasmid design of
pLenti_TRE-NPM_HygR with the regions of interest in the gray box. D. Un-transduced hiPSCs and E. hiPSCs with
pLenti_EF1a-rtTA_BsdR were used to determine the optimal concentration of HygromycinB needed to select for successfully transduced cells and 25.0 µg/mL of HygromycinB for six days was chosen.

## Slide 2
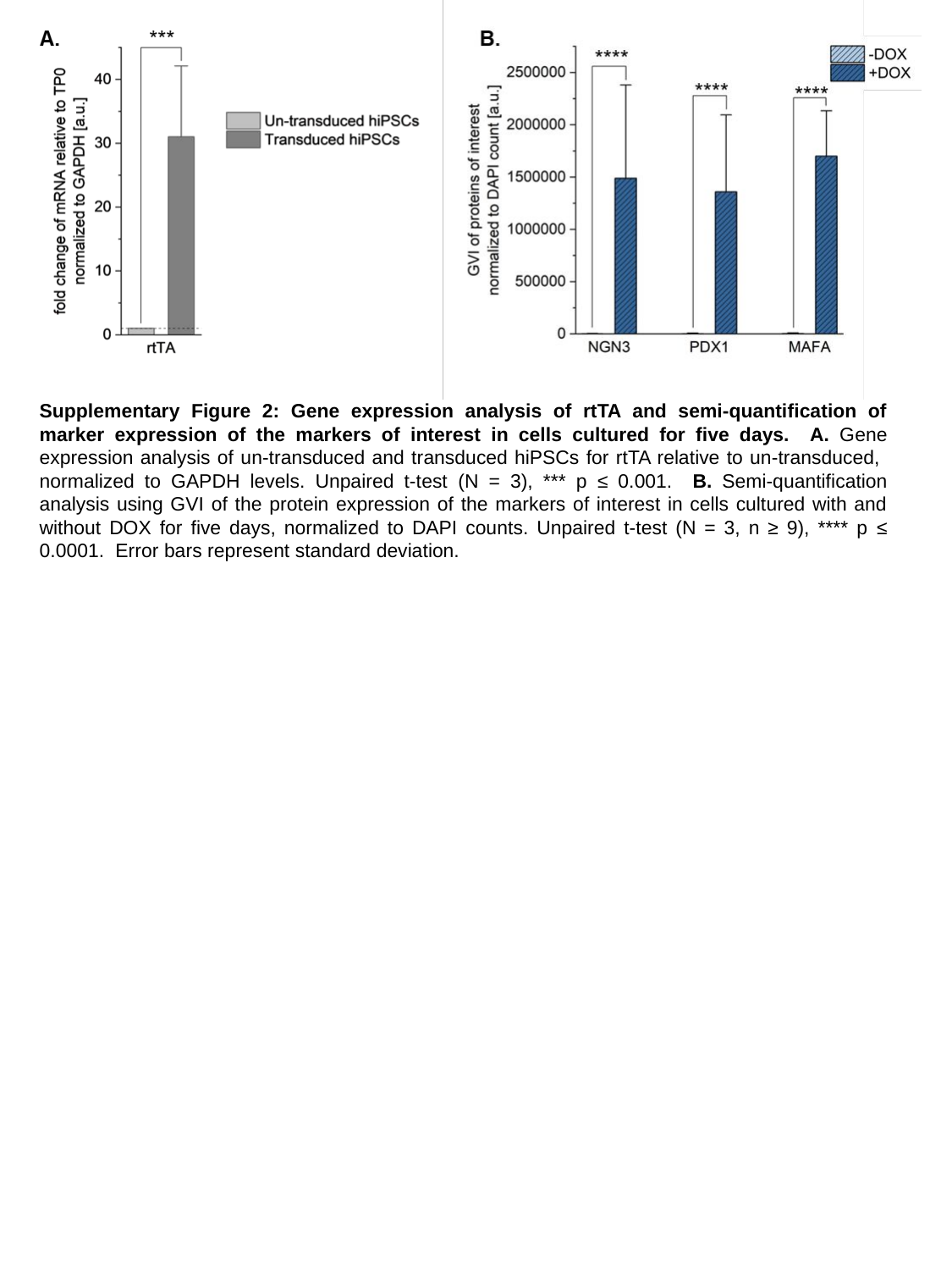

Supplementary Figure 2: Gene expression analysis of rtTA and semi-quantification of marker expression of the markers of interest in cells cultured for five days. A. Gene expression analysis of un-transduced and transduced hiPSCs for rtTA relative to un-transduced, normalized to GAPDH levels. Unpaired t-test (N = 3), *** p ≤ 0.001. B. Semi-quantification analysis using GVI of the protein expression of the markers of interest in cells cultured with and without DOX for five days, normalized to DAPI counts. Unpaired t-test (N = 3, n ≥ 9), **** p ≤ 0.0001. Error bars represent standard deviation.

## Slide 3
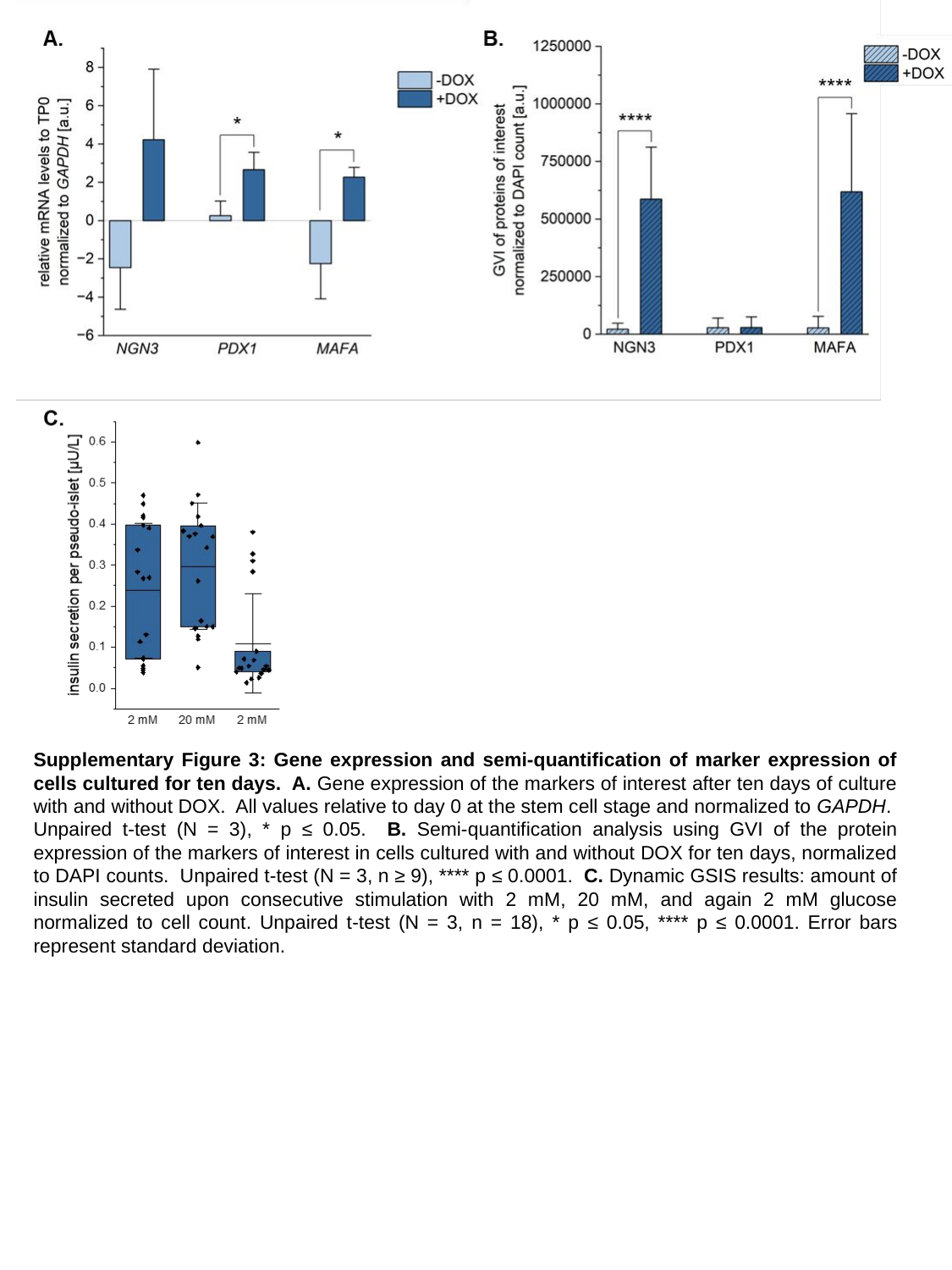

Supplementary Figure 3: Gene expression and semi-quantification of marker expression of cells cultured for ten days. A. Gene expression of the markers of interest after ten days of culture with and without DOX. All values relative to day 0 at the stem cell stage and normalized to GAPDH.
Unpaired t-test (N = 3), * p ≤ 0.05. B. Semi-quantification analysis using GVI of the protein expression of the markers of interest in cells cultured with and without DOX for ten days, normalized to DAPI counts. Unpaired t-test (N = 3, n ≥ 9), **** p ≤ 0.0001. C. Dynamic GSIS results: amount of insulin secreted upon consecutive stimulation with 2 mM, 20 mM, and again 2 mM glucose normalized to cell count. Unpaired t-test (N = 3, n = 18), * p ≤ 0.05, **** p ≤ 0.0001. Error bars represent standard deviation.

## Slide 4
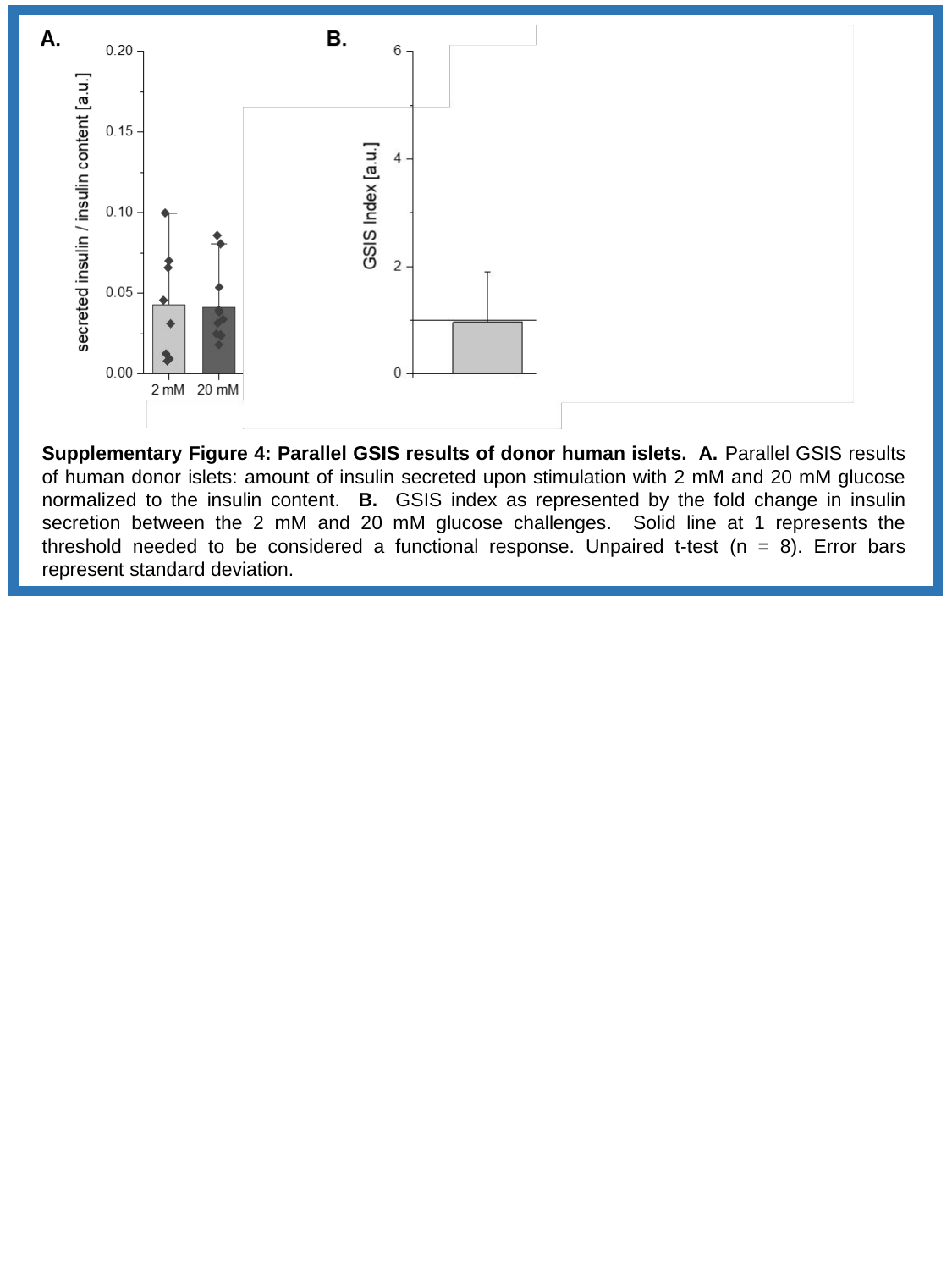

Supplementary Figure 4: Parallel GSIS results of donor human islets. A. Parallel GSIS results of human donor islets: amount of insulin secreted upon stimulation with 2 mM and 20 mM glucose normalized to the insulin content. B. GSIS index as represented by the fold change in insulin secretion between the 2 mM and 20 mM glucose challenges. Solid line at 1 represents the threshold needed to be considered a functional response. Unpaired t-test (n = 8). Error bars represent standard deviation.

## Slide 5
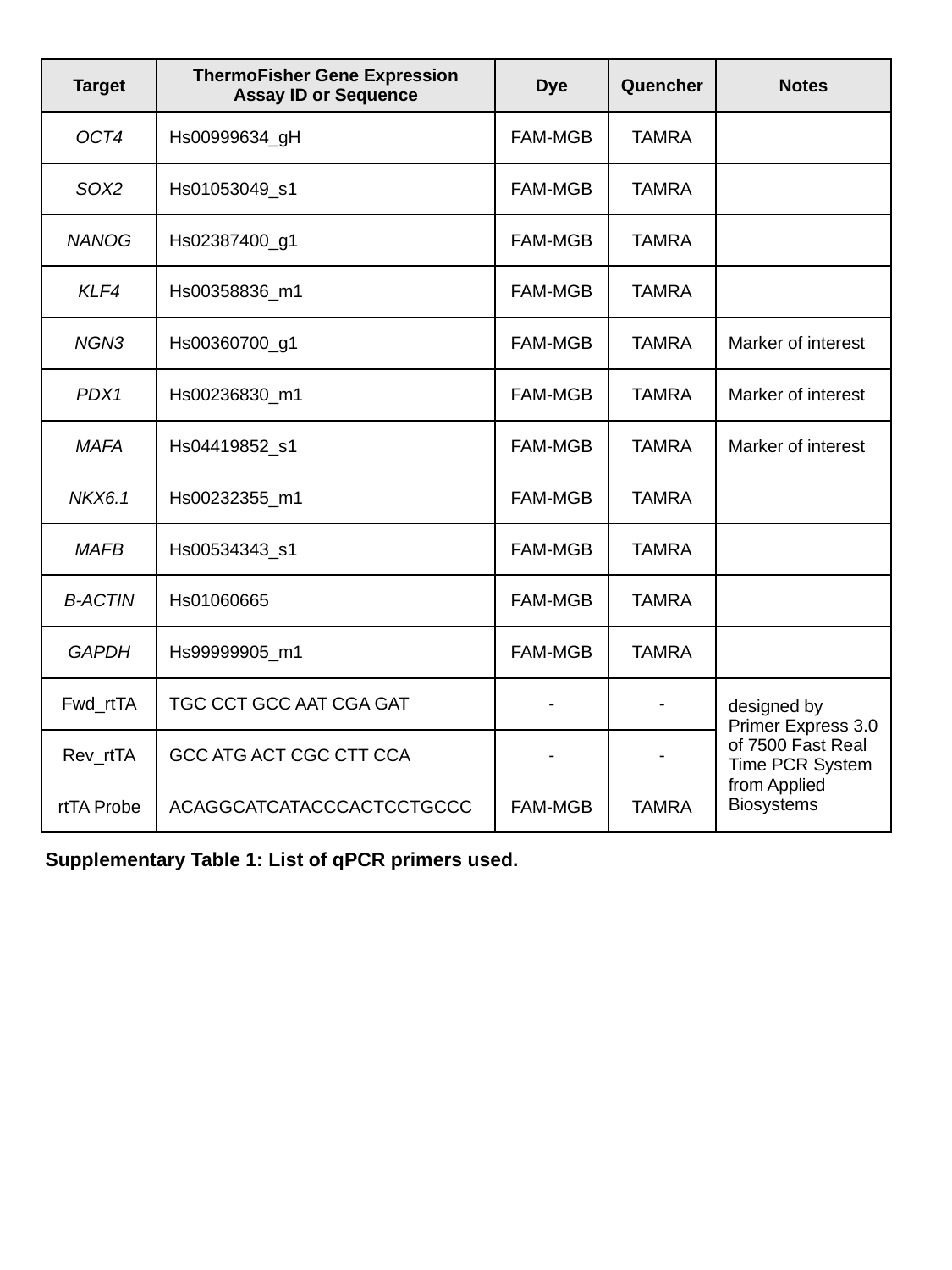

| Target | ThermoFisher Gene Expression Assay ID or Sequence | Dye | Quencher | Notes |
| --- | --- | --- | --- | --- |
| Oct4 | Hs00999634\_gH | FAM-MGB | TAMRA | |
| Sox2 | Hs01053049\_s1 | FAM-MGB | TAMRA | |
| Nanog | Hs02387400\_g1 | FAM-MGB | TAMRA | |
| Klf4 | Hs00358836\_m1 | FAM-MGB | TAMRA | |
| Ngn3 | Hs00360700\_g1 | FAM-MGB | TAMRA | Marker of interest |
| Pdx1 | Hs00236830\_m1 | FAM-MGB | TAMRA | Marker of interest |
| MafA | Hs04419852\_s1 | FAM-MGB | TAMRA | Marker of interest |
| Nkx6.1 | Hs00232355\_m1 | FAM-MGB | TAMRA | |
| MafB | Hs00534343\_s1 | FAM-MGB | TAMRA | |
| β-actin | Hs01060665 | FAM-MGB | TAMRA | |
| GAPDH | Hs99999905\_m1 | FAM-MGB | TAMRA | |
| Fwd\_rtTA | TGC CCT GCC AAT CGA GAT | - | - | designed by Primer Express 3.0 of 7500 Fast Real Time PCR System from Applied Biosystems |
| Rev\_rtTA | GCC ATG ACT CGC CTT CCA | - | - | |
| rtTA Probe | ACAGGCATCATACCCACTCCTGCCC | FAM-MGB | TAMRA | |
Supplementary Table 1: List of qPCR primers used.

## Slide 6
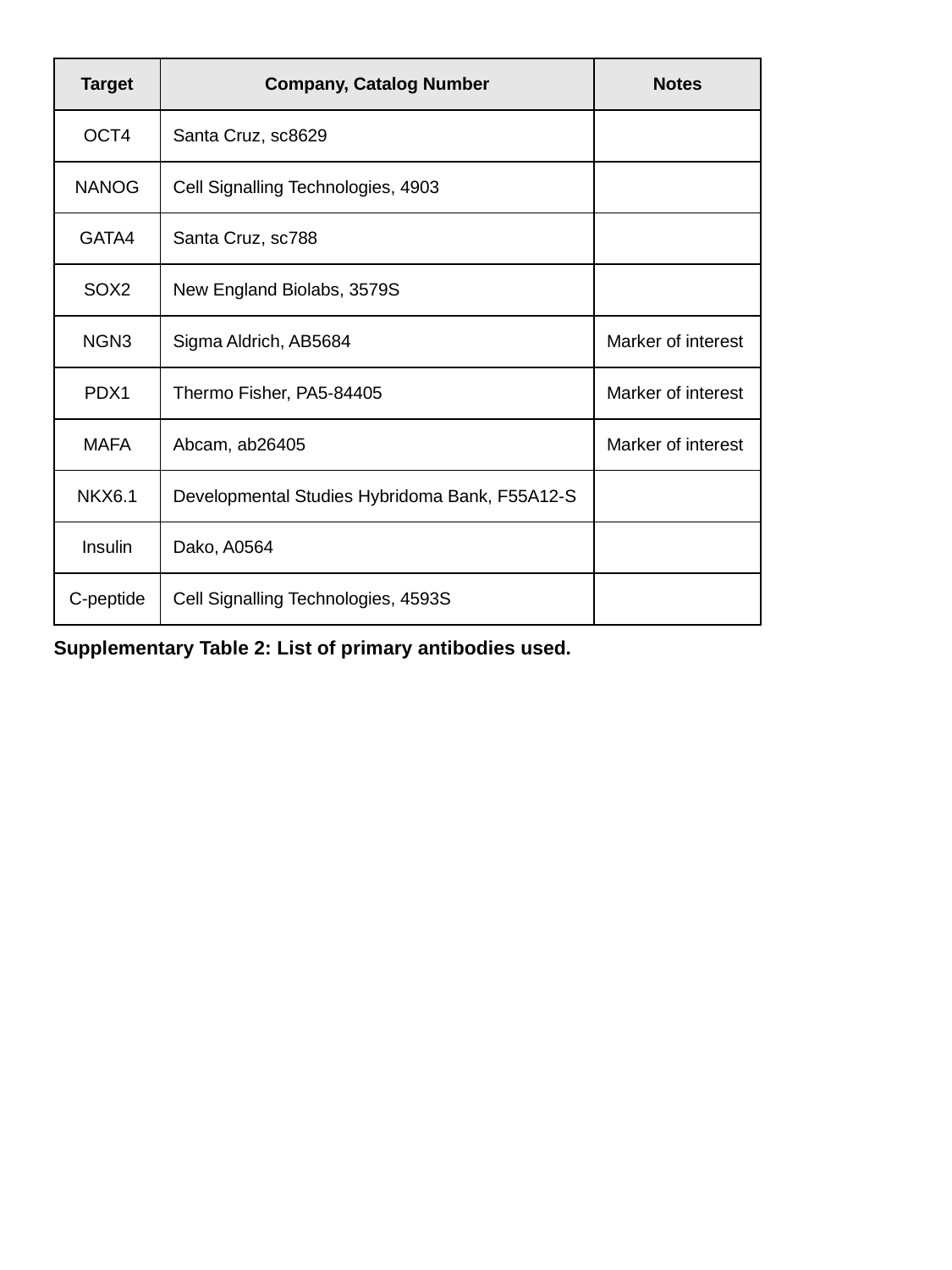

| Target | Company, Catalog Number | Notes |
| --- | --- | --- |
| OCT4 | Santa Cruz, sc8629 | |
| NANOG | Cell Signalling Technologies, 4903 | |
| GATA4 | Santa Cruz, sc788 | |
| SOX2 | New England Biolabs, 3579S | |
| NGN3 | Sigma Aldrich, AB5684 | Marker of interest |
| PDX1 | Thermo Fisher, PA5-84405 | Marker of interest |
| MAFA | Abcam, ab26405 | Marker of interest |
| NKX6.1 | Developmental Studies Hybridoma Bank, F55A12-S | |
| Insulin | Dako, A0564 | |
| C-peptide | Cell Signalling Technologies, 4593S | |
Supplementary Table 2: List of primary antibodies used.

## Slide 7
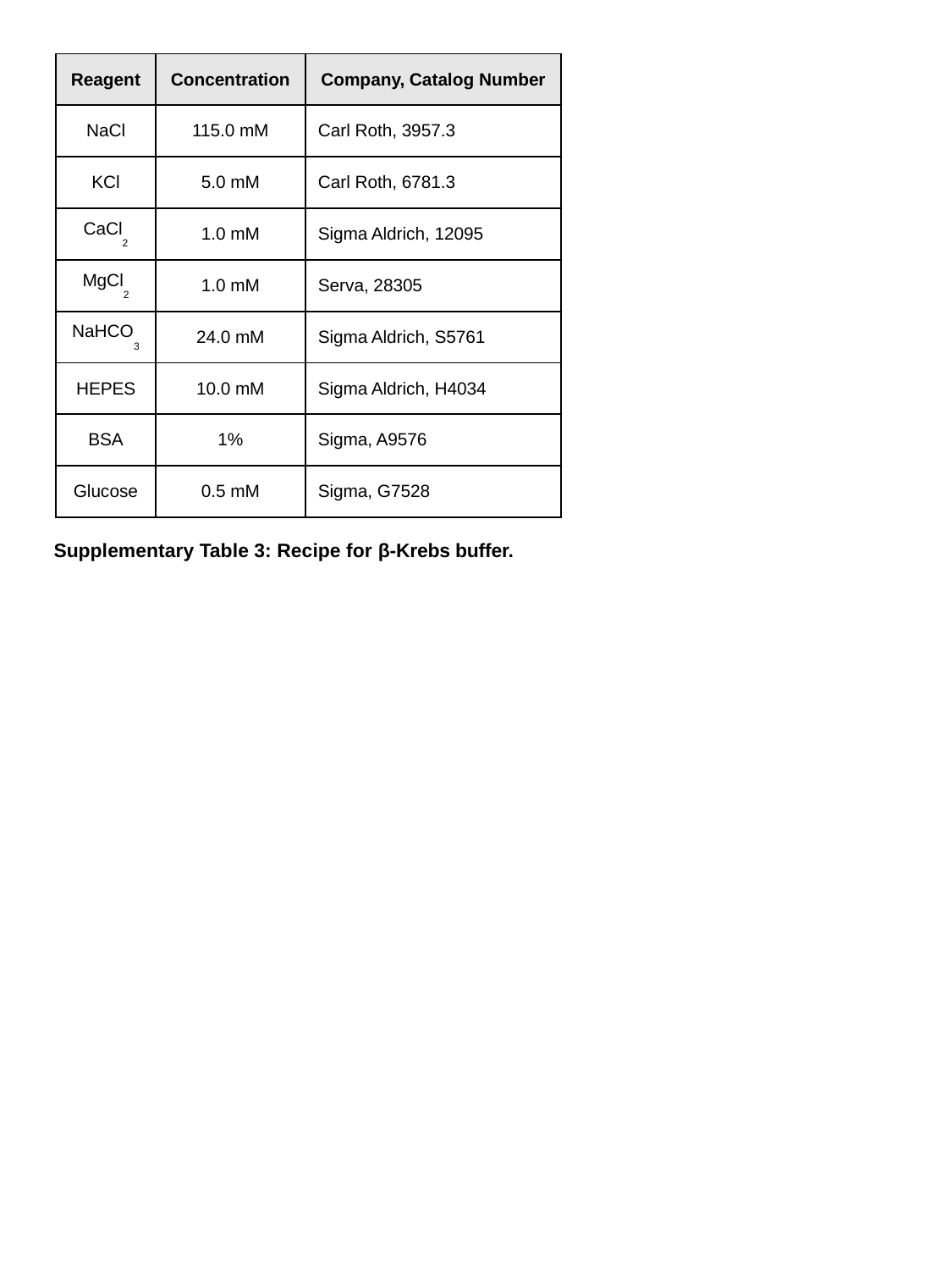

| Reagent | Concentration | Company, Catalog Number |
| --- | --- | --- |
| NaCl | 115.0 mM | Carl Roth, 3957.3 |
| KCl | 5.0 mM | Carl Roth, 6781.3 |
| CaCl2 | 1.0 mM | Sigma Aldrich, 12095 |
| MgCl2 | 1.0 mM | Serva, 28305 |
| NaHCO3 | 24.0 mM | Sigma Aldrich, S5761 |
| HEPES | 10.0 mM | Sigma Aldrich, H4034 |
| BSA | 1% | Sigma, A9576 |
| Glucose | 0.5 mM | Sigma, G7528 |
Supplementary Table 3: Recipe for β-Krebs buffer.
